# A Historical Overview on the Role of Hepatitis B and C Viruses as Aetiological Factors for Hepatocellular Carcinoma

**DOI:** 10.3390/cancers15082388

**Published:** 2023-04-20

**Authors:** Tommaso Stroffolini, Giacomo Stroffolini

**Affiliations:** 1 Department of Tropical and Infectious Diseases, Policlinico Umberto I, 00161 Rome, Italy; tommaso.stroffolini@hotmail.it; 2 Department of Infectious-Tropical Diseases and Microbiology, IRCCS Sacro Cuore Don Calabria Hospital, Via Don A. Sempreboni, 5, Negrar, 37024 Verona, Italy

**Keywords:** HBV, HCV, HCC, risk factors, prevention

## Abstract

**Simple Summary:**

Hepatitis viruses are key actors in the development of hepatocellular carcinoma (HCC), one of the leading causes of death in cancer. From the discovery of hepatitis viruses, many steps in this research field have been made. In this expert narrative review, the pathway leading to the actual public health figures is put in a historical perspective, and future scenarios are imagined for the coming years based on new technology and biological knowledge. Understanding the historical context of the role of hepatitis viruses in the development of HCC is important for improving our comprehension of the mechanisms by which these viruses contribute to cancer development, expanding strategies for prevention and treatment, and improving public health policies to reduce the burden of HCC in populations with high rates of viral hepatitis.

**Abstract:**

Hepatitis B virus (HBV) and hepatitis C virus (HCV) are the leading cause of hepatocellular carcinoma (HCC) worldwide. Currently, HBV-related HCC predominates in Sub-Saharan Africa and South-East-Asia, while HCV-related HCC predominates in northern Africa and in the western world. Liver cirrhosis is the underlying condition in most HBV cases and in nearly all HCV cases. Several cofactors, viral and non-viral, play a role in the progression toward HCC: dual HBV/HCV infection, HDV, HIV, alcohol intake, smoking, diabetes mellitus, obesity, and NAFLD/NASH. HBV vaccine is effective in preventing both infection and HCC; antiviral drugs may suppress HBV replication and eradicate HCV infection, halting progression to HCC. Inequalities exist between high- and low-income countries with respect to vaccine availability and access to antivirals. These factors represent barriers to the control of HCC incidence. Lack of an effective vaccine against HCV is also a serious barrier to HCV elimination and HCC prevention. The most crucial steps and knowledge that have arisen over time on the association between the two major hepatotropic viruses and HCC are discussed in this historical review.

## 1. Introduction

Liver cancer ranks as third in what pertains to mortality among all cancers with 780,000 reported deaths in 2018 [1]. Currently, 75–80% of liver cancer cases are estimated to be due to hepatocellular carcinoma (HCC) [2]. Hepatitis B virus (HBV) and hepatitis C virus (HCV) have played and continue to play a major role as aetiological factors for HCC. In HBV-related HCC, cirrhosis might be absent in up to one third of the cases; conversely, it is present in almost all HCV-related HCCs [3]. Nowadays, the knowledge that a virus might cause liver cancer is a well-recognized fact; that was not the case until the beginning of the 1980s as this relationship was veiled and unexplored [4]. Generally speaking, the geographical incidence of HCC may vary according to the endemic level of these two viruses, the availability and supply of vaccines against HBV, the access to antiviral treatment for both viruses, and the increasing role of lifestyle factors. In this review, we describe the historical knowledge regarding the relationship between hepatotropic viruses and HCC and the potential future scenario of HCC due to the growing role of non-viral factors.

## 2. Hepatitis B

Not more than 58 years ago, hepatitis B was a disease by name alone. Diagnostic assays, treatments, and effective preventive measures were absent. In 1965, Baruch Blumberg, while studying “yellow jaundice”, discovered a surface antigen for hepatitis B in the blood of an Australian aborigine, hence initially called the “Australian antigen” [5]. Later, he developed the diagnostic test and the original vaccine. For these outstanding results, he received the Nobel Prize in 1976.

In 1980, a placebo-controlled trial of an inactivated hepatitis B vaccine in high-risk homosexual men showed an unequivocal efficacy of reducing the incidence of HBV infection by 93% [6]. It represented one of the biggest achievements in the medical research during the last decades of the so called “short century”. One year later, the first compelling epidemiological evidence for the causative role of HBV infection in HCC development was provided by a very large prospective study in Taiwan [4]. The relative risk of HCC was more than 200-fold higher in HBV-infected individuals than in uninfected controls. This unequivocal association also supported that the recently developed HBV vaccine would possibly prevent HBV-related HCC, therefore representing the first cancer vaccine. This major achievement set the stage for the universal HBV vaccination of infants in Taiwan in 1984. The campaign was progressively extended to all elementary school children by 1990. The campaign resulted effective in preventing HCC during the 20 years after its launch: a 75% reduction in the incidence of HCC among 6–9 years old children was observed in a subsequent study [7]. Similarly, after universal HBV vaccination was launched in Alaska, the incidence of HCC in persons <20 years decreased from 3/100,000 in 1984–1988 to zero in 1995–1999, and no HCC cases have occurred since 1999 [8]. To sum it up, there is no doubt HB vaccine has been proven effective in preventing HCC.

Several cofactors, viral and non-viral, have been identified to be able to enhance progression to HCC in HBsAg positive subjects. 

### 2.1. Viral Cofactors

#### 2.1.1. HCV Coinfection

The availability of an ELISA method for anti-HCV detection at the beginning of the 1990s allowed the evidence for the additive interaction of the contemporary presence of both HBsAg and anti-HCV positivity on the risk of developing HCC [9]. Additive interaction means that the observed joint effect of two factors on the disease incidence (in this case, HCC) exceeds the sum of the effect of exposure to each single factor minus 1. In fact, compared to subjects negative for both viruses (reference category), the risk of HCC was 13.3-fold higher in HBsAg-positive subjects alone and 21.3-fold higher in those anti-HCV-positive alone, but it was 77.0-fold higher when both exposures were present (Table 1).

The effect of dual HBV/HCV infection was confirmed at the end of the 1990s in a prospective study of patients with compensated cirrhosis [10]. The incidence rate of HCC × 100 person/years of follow-up was 2.0% in subjects HBsAg+/anti-HCV− and 3.7% in the HBsAg−/Anti-HCV+, but it was 6.4% in the HBsAg+/Anti-HCV+ group. 

#### 2.1.2. HDV Coinfection

Coinfection with hepatitis Delta virus [11], a virus that only exists in the setting of HBV infection as a co-existing infection because of its reliance on HBV for propagation [12], generates a 3.2-fold increased risk for HCC [13]. Some studies have suggested even the possibility of a direct HDV oncogenic activity [12]. 

### 2.2. Non-Viral Cofactors

An additive and multiplicative interaction of the contemporary presence of both HBsAg positivity and smoking on the risk of HCC has been demonstrated. Multiplicative interaction means that the observed joint effect of two factors on the disease incidence exceeds the product of the effect of exposure to each single factor. In fact, compared to HBsAg−/non-smokers (reference category), the adjusted ORs were 1.25 for HBsAg−/ever-smokers, 7.66 for HBsAg+/never-smokers, and 15.68 for HBsAg+/ever-smokers [14] (Table 2).

Moreover, alcohol intake >60 g/day increases nearly two-fold the risk of HCC in HBV-positive subjects [15]. Additionally, metabolic risk factors play an important role in HCC development in HBV-positive subjects, as demonstrated by the evidence that high body mass index (BMI) increases the risk of HCC [16]. Importantly, a metanalysis that included 11,000 subjects with chronic hepatitis B (CHB) and diabetes mellitus type II clearly demonstrated the increased risk of HCC for the combination of these conditions (pooled HR = 1.77 95% CI: 1.28–2.47) [17]. 

Occult hepatitis B infection (OBI) is a condition in which HBV DNA can be found in the liver tissue and occasionally even in the serum of subjects, in the absence of circulating HBsAg [18]. That condition has been described as the persistence of HBV infection in HBsAg-negative subjects that also suffered from HCC [19]. However, a direct association between the status of OBI and the risk HCC has not been definitively established [20]. Accordingly, the final report of the Taormina meeting on OBI suggested further studies to confirm this theoretically possible association [21]. More recently, a study from Korea [22] has shown that among isolated anti-HBc-positive patients, the risk of liver cancer mortality was significantly higher in those with high fibrosis-4 (FIB4) scores compared to patients unexposed to HBV (adjusted HR 72.66, CI 95% = 36.96–142.86). 

## 3. HBV Carcinogenesis

The presence of integrated HBV DNA into the hepatocytes represents the key point for HBV-related liver carcinogenesis. In 1981, the presence of liver integrated HBV DNA sequences was firstly described by applying a hybridisation technique [23]. This evidence allowed the biological plausibility to the epidemiological causal association linking HBV and HCC that was published a month later in the same journal [4]. One year later, integrated HBV DNA sequences were found in the vast majority of analysed HCC cases, confirming this causal relationship [24]. Further evidence for the association between the presence of HBV DNA in the liver and HCC was provided 9 years later among children with HCC in Taiwan [25]. Almost all children with HCC were confirmed HBsAg+ and had HBV DNA integrated in the genome of their neoplastic tissue [26]. 

After exposure to HBV, HBV DNA is transformed in covalently closed circular (ccc) DNA, which is integrated in the host genome inside the hepatocyte nucleus [27].

During the last decades, several studies have classified the various direct and indirect mechanisms promoting hepatocellular carcinogenesis. A recently published review article summarizes elegantly the advances in the knowledge of the molecular profiles of HBV–HCC interactions and the altered molecular pathways modifying the microenvironment and leading to DNA damage [28].

## 4. Prevention of HBV-Related HCC

### 4.1. Primary Prevention (For Subjects That Are Still Susceptible to HBV)

As already pointed out, vaccination has proven very effective in preventing HCC [7,8]. Very large and ambitious vaccination programs against HBV are taking place in several countries. A recent report from Italy analysing acute HBV epidemiological trends over time has shown that the WHO goal of controlling HBV infection is close to being reached for the first time in Europe; zero HBV infections in the age group 0–14 years old, 0.1 × 100,000 population in the age group 15–24, and 0.5 × 100,000 population in subjects older than 24 years of age were reported [29]. The key explanation for this outstanding result comes as a consequence of the vaccination policy adopted in this country: the combined compulsory immunisation of 3-month-old infants and of the 12-year-old subjects (limited to the first 12 years of the campaign for the latter category) has generated an early immune cohort of youths not at risk of acquiring HBV infection. As a result, all subjects below the age of 42 years are currently vaccinated against HBV in Italy. 

Unfortunately, in some endemic HBV areas, such as Sub-Saharan Africa and South East Asia, HB massive vaccination campaigns are not in place, and vaccine coverage is suboptimal [30,31].

This represents one of the most serious barriers against the WHO goal for worldwide HBV infection control by 2030. 

Recommendations to injecting drug users (IDUs) in order to avoid the sharing of needles and syringes remains of paramount importance (Table 3).

Drug use still represents a major source of HBV infection in the USA, despite an overall decline in acute HBV attributable to successful vaccination campaigns [32]. In fact, the proportion of people reporting intravenous drug use as source of HBV exposure in the USA nearly doubled from 35.3% in 2001–2006 to 58.4% in 2007–2018 [33]. However, these different prevalences did not reach a statistically significant level, probably for the small number of persons in each year-grouping.

### 4.2. Secondary Prevention (For Subjects with Chronic HBV Infection)

Antiviral treatment with nucleos(t)ides analogues (NAs) is an important tool to prevent HCC in subjects with HBV infection, as these drugs are able to suppress viral replication. NAs became available at the beginning of the 2000s. However, first and second-generation NAs presented a moderate to high risk of treatment failure. In the second half of the 2000s, the advent of safer and more effective third generation NAs (namely, entecavir and tenofovir disoproxil fumarate) changed the overall picture, especially in the case of therapy started before the development of cirrhosis [34]. HCC mortality could be prevented in the majority of cases, given that treatment with NAs was provided for more than five years [35]. Unfortunately, both drugs are unable to eliminate the HBV DNA integrated sequences from infected hepatocytes [34]. 

The combination of new drugs that eliminate or functionally suppress and inactivate the genomic HBV reservoirs (cccDNA and integrated HBV DNA) may represent a further step for HBV control and morbidity reduction. Several new drugs that target distinct pathways of the HBV life cycle have been developed [36]. At least some of them are expected to enter clinical practice. That is of outmost importance as, more recently, a study coupling deep molecular biology and tissue analysis also found that a small proportion of the cccDNA reservoir is constantly replenished by continued low-level HBV replication even after prolonged treatment with NAs [37].

Other secondary preventive measures are weight control and the avoidance of alcohol intake and smoking (Table 3).

## 5. Hepatitis C

At the end of the 1980s, the cloning of the non-A, non-B hepatitis virus (now called hepatitis C virus) genome represented a milestone in the field of liver disease knowledge [38]. The development of a test for the detection of circulating HCV antibodies provided an important diagnostic tool and evidence of the role of HCV infection in chronic liver diseases, including HCC [39]. The epidemiological association between HCV and HCC was firstly demonstrated in a case control study performed in the USA in 1990: HCV positivity was 10.5-fold (CI 95%: 3.5–31.3) more likely detected in HCC cases than in the control group [40]. The almost universal presence of underlying liver cirrhosis in HCV-related HCC rose the question whether HCV infection was a risk factor for HCC because of the underlying cirrhosis or it increased the risk of HCC in cirrhotic subjects. The answer came from a case-control study performed in 1991 [41]. Subjects with HCC and cirrhosis were two-fold (CI 95% 1.3–3–2) more likely to be HCV-positive than those with cirrhosis alone. It provided evidence that HCV infection is an independent risk factor for developing HCC, both because it induces cirrhosis and increases the risk in patients with cirrhosis. Several subsequent studies have confirmed this epidemiological association that is now a well-established fact [3]. Currently, HCV-attributable HCC cases are the majority in western countries [42], with an increasing risk as cirrhosis progresses. 

## 6. Risk Factors for HCC in HCV Patients C

### 6.1. Viral Cofactors

Co-infection with HBV and OBI has been previously discussed (see HBV paragraph). Other viral cofactors play a major role. Importantly, HIV co-infection in HCV subjects generates the progression of fibrosis and cirrhosis [43,44], increasing the risk of HCC compared to HCV mono-infected subjects [45]. However, the current effective antiretroviral therapy (ART) for HIV and the availability of an effective antiviral treatment for HCV have generated a nearly similar HCC incidence in HIV/HCV-infected individuals and HCV mono-infected ones [46]. 

To date, no other major or minor viral infections have been found to interact with HCV for the risk of HCC.

### 6.2. Non-Viral Cofactors

As for HBV, and even for HCV, cofactors such as those related to lifestyle are associated with an increased risk of HCC. The relative risk of HCC in HCV-positive smokers resulted higher than in non-smokers (23.0 vs. 7.9) [47]. Evidence suggests that diabetes mellitus also increases the risk of HCC in HCV-infected individuals [48]. Moreover, steatosis is an independent risk factor associated with fibrosis progression in HCV individuals leading to a higher risk of HCC [49].

On the other hand, alcohol consumption has been shown to increase both the rate of fibrosis [50] and accelerate carcinogenesis [51] in HCV-positive patients. Accordingly, a case-control study showed a dose-dependent effect between HCV positivity and increasing doses of daily alcohol intake on the risk of HCC: the OR for the different level of daily alcohol intake was 26.1 (CI 95% 12.6–54.0) for 0–40 g, 62.6 (CI 23.3–168) for 41–80 g, and peaked at 126 (42.8–379) for >80 g [52] (Table 4).

A prospective study among HCV-positive patients showed a 5-year cumulative incidence of 10.6% for HCC among abstaining subjects, increasing to 23.8% among those with a median alcohol intake of 15 g/day [53]. The biological plausibility of these epidemiological findings was provided by further data: alcohol consumption impairs immune response [54] and may promote apoptosis in hepatocytes infected with HCV [55]. The synergism between HCV and alcohol intake is very relevant in terms of public health in countries where both exposures are frequent, such as Eastern Europe and the Mediterranean area. Interestingly, in an Italian study in 2014, as many as 41.1% of subjects with alcohol-related chronic liver disease were also HCV-positive, stressing the importance of the co-frequency of these factors [56]. 

## 7. HCV Carcinogenesis

Differently from what has been described in HBV viral infection, HCV does not integrate into the host. HCV carcinogenesis is promoted by a chronic inflammatory process evolving in fibrosis [57]. Proliferative changes in fibrotic tissue, due to the ongoing inflammation, generate repeated cycles of cell death and tissue regeneration [58]. Repeated cell cycles may be associated with mutations that are able to transform hepatocytes into malignant cells. Other factors are discussed in the Section 9.

## 8. Prevention of HCV-Related HCC

### 8.1. Primary Prevention (For Subjects That Are Still Susceptible to HCV)

The lack of an effective vaccine against HCV represents a major barrier against the control of the infection. Other than blood screening for transfusion, the main primary prevention is avoidance of sharing glass syringes equipment for IDUs, who are at high risk of exposure to HCV in the western world (OR 30.2; CI 95%, 12.7–71.9, adjusted for the confounding effect of age, sex, years of education, and past blood transfusion by multiple logistic regression analysis) [59].

### 8.2. Secondary Prevention (For Subjects Who Are Infected with HCV)

As already pointed out, HB vaccination is very important for HCV-positive subjects who are still susceptible to HBV because of the additive effect on the risk of HCC when both infections are present [9]. Unfortunately, this preventive measure is scarcely applied. A recent survey in Italy has shown that only 20.5% (72/352) of cirrhotic HCV-positive patients result to have received HB vaccination [60]. 

Antiviral therapy represents the most effective intervention for preventing HCV progression and complications, including HCC. The goal of antiviral therapy is the so-called sustained virological response (SVR) (meaning the eradication of the virus). Interferon (IFN) based therapy has been used for more than two decades. The drug could lower HCC risk trough anti fibrotic effect by reducing inflammation and trough antineoplastic properties [61]. Data coming from real life use showed that IFN therapy resulted effective in reducing HCC occurrence: among 920 Caucasian cirrhotic cases during a mean follow up of 96.1 months, the incidence rate of HCC × 100/persons year was 0.66 in SVR subjects and 2.10 in those not reaching SVR (*p* < 0.001). Moreover, the risk of HCC was 2.66-fold (CI 95%, 1.13–5.97) higher for those not reaching SVR [62]. A meta-analysis performed at the beginning of the 2010 concluded that IFN therapy was effective in preventing HCC in people who achieved SVR [63]. Unfortunately, IFN therapy was often poorly tolerated due to relevant side effects, and only a limited proportion of subjects with liver cirrhosis completed therapy and reached SVR (<20%) [64]. Since the year 2014, direct antiviral agents (DAAs) became available. These drugs are characterized by a very safe and effective profile, achieving SVR rates >95%, even in cirrhotic patients [65]. DAAs therapy has been demonstrated effective in lowering the incidence of HCC in SVR subjects as compared to those not reaching SVR. In an American retrospective study analysing data about this population, the incidence of HCC was reduced by 83.5% in SVR subjects [66]; in a prospective Italian study, the incidence rate of HCC at one year was 2.6% in patients achieving SVR and 8.0% in patients not achieving SVR [67]. However, cirrhotic subjects who achieve SVR are still at risk of developing HCC, even if at a lower rate, as the presence of cirrhosis is an independent risk factor for HCC. Consequently, ultrasound surveillance is recommended even after HCV eradication. 

Other relevant measures for secondary prevention are weight control and the avoidance of alcohol intake and smoking (Table 5). 

## 9. Future Scenarios

**Figure 1:**
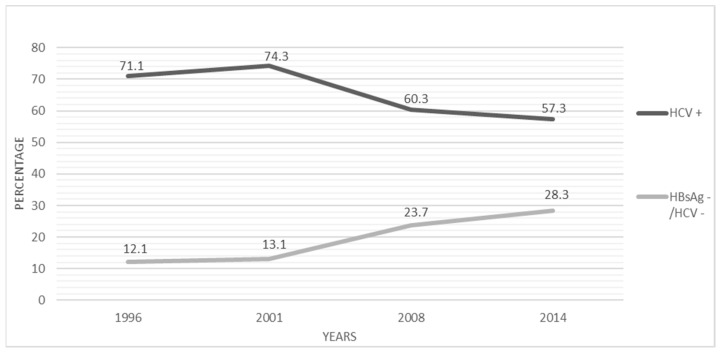
Temporal trends of the proportion (%) of anti-HCV positivity and hepatitis viruses negativity among HCC patients in Italy, 1996–2014. Adapted from Reference 68. (X^2^ for linear trend = *p* < 0.001).

HBV and HCV infections have been and still continue to be major causes of HCC within a changing geographical picture: HBV predominates in Sub-Saharan Africa and Southeast Asia, while HCV is the bigger risk factor in northern Africa and in the so-called western world. Several interventions have reduced the endemic level of the two viruses over the last decades worldwide: vaccination campaigns against HBV, improved sanitary conditions, information against sharing drug use equipment and unsafe sexual behaviours, blood transfusion screening, and availability of effective antiviral drugs. These factors have ultimately generated a decreased rate of viral-related HCCs, especially in high-income countries. In Italy, the proportion of HCV-related HCC cases significantly decreased over nearly 3 decades (1996–2014) from 71.3% to 53.2%; conversely, the proportion of HCV-negative/HBsAg-negative HCC cases increased from 12.1% to 28.3% (<0.01) (Figure 1).

The proportion of HBV-related HCC cases remained stable (around 13%) during the same period [68]. In agreement with these findings, a recent survey has shown a significant increase over time in the proportion of HCC cases associated with metabolic associated fatty liver disease (MAFLD) as a single aetiology, increasing from 3.6% in 2002–2003 to 28.9% in 2018–2019 (*p* < 0.001) [69]. MAFLD represents a new inclusive definition of the whole spectrum of liver disease associated to metabolic disorders; it is defined as having hepatic steatosis and any 3 of the following: overweight/obesity, diabetes mellitus, metabolic dysregulation. Conversely, NAFLD is generally defined as having fatty liver disease in the absence of known causes such as alcohol, HBV or HCV; it is now the leading cause of HCC occurring in the absence of significant fibrosis [70]. Up to 50% of NAFLD subjects may evolve to HCC without underlying cirrhosis [71]. A very high proportion of 65% has been recently observed in a surgical referral centre in France, despite these findings likely being affected by a referral bias [72].

Broadening our vision, a new world of evidence coming from deep nucleic acid analysis, molecular biology, and omics methods coupled with artificial intelligence may trace unexplored trajectories for the interpretation that has been conceived over the last decades for HCC carcinogenesis and help elucidate the role of hepatotropic major viruses [73]. In particular, epigenetic factors, microRNA (miRNA) and mRNA regulatory pathways, and immune cell subsets derived interactions triggered by these two major viruses should be taken into account for the development of future preventive and therapeutic strategies [74,75]. However, detailed mechanisms of these type of interactions, especially changes in DNA methylation and gene expression between the two types of virus-related HCC, and their contributions to the HCC development, metastasis, and recurrence remain largely unknown [76].

Epigenetic mechanisms are dynamic molecular processes that regulate gene expression without altering the host DNA, acting by modifying the host chromatin structure via covalent post-translational histone modifications, changing the DNA methylation status, expression of non-coding RNAs such as miRNAs and long noncoding RNAs, and altering the spatial, three-dimensional organization of the chromatin of the virus-infected cell [77]. A recent review described the main evidence in support of the de-regulation of epigenetic mechanisms in the HBV-infected/-replicating hepatocyte and their contribution to hepatocyte transformation [78]. In contrast to genetic mutations which are permanent, epigenetic alterations are dynamic and possibly reversible. For this reason, patients with and without an SVR to DAAs therapy and epigenetic and gene expression alterations associated with risk for HCC have been investigated [79]. Importantly, the epigenetic changes induced following infection persisted as an “epigenetic signature” even after virus eradication by DAAs treatment [80], providing interesting insights in the unresolved matter of quantifying HCC risk after viral eradication. Other data coming from metabolomic and proteomics analysis have confirmed the relevance of coupling methodologies and provide biological explanations of clinical and epidemiological occurrences applying the principles of translational medicine [81].

These data provide evidence for the importance of such new methodologies applied to the HCV-HBV interactions and the development of HCC. Importantly, these findings may help in developing new primary and secondary preventive approaches and tailoring strategies both at the public health and patient level.

## 10. Conclusions

In conclusion, the findings presented in this historical overview suggest that in the western world NAFLD might represent the leading cause of HCC in the years to come, reflecting the lifestyle changes that are typical to this area and society. Concerns arise for the disparities between high and low-middle income countries where vaccination campaigns against HBV and access to effective antiviral therapy are scarce. That represents a serious barrier against the control of the progression to HCC. Finally, the absence of an effective vaccine against HCV should be stressed, being that this intervention is very important for the control of this infection, even in high-income countries. 

## Figures and Tables

**Table 1:** Evidence for the additive interaction of the contemporary presence of HBsAg and anti-HCV positivity on the risk of HCC development (adapted from [9]).

HBsAg	Anti-HCV	OR (CI 95%)
−	−	1
+	−	13.3 (5.5–32.2)
−	+	21.3 (8.8–51.5)
+	+	77.0 (3.8–142.1)

**Table 2:** Evidence for the additive and multiplicative interaction of the contemporary presence of smoking and HBV infection on the risk of HCC development (adapted from [14]).

Ever-Smoker	HBsAg+	OR (CI 95%)
No	No	1
No	Yes	7.66 (6.05–9.71)
Yes	No	1.25 (1.03–1.52)
Yes	Yes	15.68 (12.06–20.39)

**Table 3:** Preventive measures for HBV-related HCC. IG: immunoglobulins; IDU: injecting drugs users; NAs: Nucleo(s)tides analogous.

Primary prevention	- Vaccine and IG for neonates of HBsAg+ mothers - Vaccine alone for other age groups - Avoidance of sharing equipment in IDU
Secondary prevention	- NAs antiviral therapy - Smoking avoidance - Alcohol intake avoidance - Weight control, Diet, Exercise

**Table 4:** Evidence for the dose-dependent effect of alcohol intake in HCV-positive subjects on the risk of HCC (adapted from [52]).

Alcohol Intake (g/day)	O.R. (95% CI) for HCC in HCV-Positive Subjects
0–40	26.1 (12.6–54.0)
41–80	62.6 (23.3–168.0)
>80	126.0 (42.8–379.0)

**Table 5:** Preventive measures for HCV-related HCC. IDU: injecting drugs users. DAAs: Direct Antiviral Agents.

Primary prevention	- Avoidance of sharing drug-use equipment
Secondary prevention	- HB vaccine for HCV-positive subjects still susceptible to HBV - DAAs therapy - Avoidance of alcohol intake and smoking - Weight control
